# Prediction of Clinical Deterioration in Hospitalized Adult Patients with Hematologic Malignancies Using a Neural Network Model

**DOI:** 10.1371/journal.pone.0161401

**Published:** 2016-08-17

**Authors:** Scott B. Hu, Deborah J. L. Wong, Aditi Correa, Ning Li, Jane C. Deng

**Affiliations:** 1 Division of Pulmonary/Critical Care, University of California, Los Angeles, Los Angeles, California, United States of America; 2 Division of Hematology/Oncology, University of California, Los Angeles, Los Angeles, California, United States of America; 3 Downstate College of Medicine, State University of New York, Albany, New York, United States of America; 4 Department of Biomathematics, University of California, Los Angeles, Los Angeles, California, United States of America; Universita degli Studi di Catania, ITALY

## Abstract

**Introduction:**

Clinical deterioration (ICU transfer and cardiac arrest) occurs during approximately 5–10% of hospital admissions. Existing prediction models have a high false positive rate, leading to multiple false alarms and alarm fatigue. We used routine vital signs and laboratory values obtained from the electronic medical record (EMR) along with a machine learning algorithm called a neural network to develop a prediction model that would increase the predictive accuracy and decrease false alarm rates.

**Design:**

Retrospective cohort study.

**Setting:**

The hematologic malignancy unit in an academic medical center in the United States.

**Patient Population:**

Adult patients admitted to the hematologic malignancy unit from 2009 to 2010.

**Intervention:**

None.

**Measurements and Main Results:**

Vital signs and laboratory values were obtained from the electronic medical record system and then used as predictors (features). A neural network was used to build a model to predict clinical deterioration events (ICU transfer and cardiac arrest). The performance of the neural network model was compared to the VitalPac Early Warning Score (ViEWS). Five hundred sixty five consecutive total admissions were available with 43 admissions resulting in clinical deterioration. Using simulation, the neural network outperformed the ViEWS model with a positive predictive value of 82% compared to 24%, respectively.

**Conclusion:**

We developed and tested a neural network-based prediction model for clinical deterioration in patients hospitalized in the hematologic malignancy unit. Our neural network model outperformed an existing model, substantially increasing the positive predictive value, allowing the clinician to be confident in the alarm raised. This system can be readily implemented in a real-time fashion in existing EMR systems.

## Introduction

Approximately 5–10% of hospitalized patients suffer a significant adverse event after admission, including transfer to the intensive care unit (ICU) or cardiopulmonary arrest.[1] Delays in identification of clinical deterioration along with delayed therapeutic interventions result in increased morbidity and mortality.[2–5] With increasing physician and nursing workloads and more handoffs of care, prompt recognition of a deteriorating patient has become increasingly difficult. Hence, automated systems that alert the medical staff of impending clinical deterioration may enable clinicians to intervene at an earlier time, thereby preventing an arrest or reducing the need for ICU transfer.

To address this issue, a multitude of early warning systems and scores (EWS) have been developed with the goal of identifying patients who are at risk for imminent cardiac arrest or ICU transfer. Most of these early warning scoring systems (also known as "track and trigger" systems; e.g., Modified Early Warning Score or MEWS, VitalPac EWS or ViEWS, National EWS or NEWS, Rothman index) rely heavily upon vital sign abnormalities and assessment of mental status.[6] With the increase in electronic and automated monitoring of hospitalized patients, EWS-based systems have been adopted by many hospitals in an attempt to identify patients who are deteriorating and require escalation of care. However, accurate prediction of a patient who requires impending ICU transfer is difficult, as many stable patients may reach the "trigger" threshold for an event but not ultimately require ICU transfer. As a result, current early warning system-based prediction models have good sensitivity but poor positive predictive values (e.g., 5–10%).[1, 7–13] Current clinical monitoring alarms also suffer from poor specificity, with published studies reporting false alarm rates of 70–95%, potentially resulting in alarm fatigue[10, 14–16], which has been associated with patient death.[17] Not surprisingly, subsequent studies have demonstrated that EWS-based alarms only marginally improve outcomes while substantially increasing physician and nursing workloads.[18]

Given the limitations of currently available monitoring systems, we wanted to develop a novel prediction tool based upon advanced machine learning algorithm called a neural network (or multilayer perceptron) that would utilize readily available vital signs and laboratory values that are routinely obtained during the course of a typical hospital admission.[19] Neural networks are a class of machine learning algorithms that have the ability to discern complex nonlinear patterns and have been used primarily for basic science applications such as gene identification, genetic interaction and protein structure prediction.[19, 20] In addition, mathematical modeling of biological systems have been performed in basic science, including that of tumor biology.[21, 22] We hypothesized that a neural network (NN)-based model built upon a patient's "static" characteristics (e.g., age, gender, diagnosis), coupled with "dynamic" variables (e.g., an individual's pattern of vital signs and lab results over the duration of hospitalization) would enhance predictive ability of clinical deterioration. We defined clinical deterioration as ICU transfers and cardiac arrests, given that these events are associated with increased in-hospital mortality. ICU transfers and cardiac arrest therefore served as objective outcomes for model training and for prediction, similar to prior studies.[1, 7, 9] We determined whether our neural network-based prediction model would have increased sensitivity and higher positive predictive value compared to existing scoring systems. For the initial phase of our study, we chose to analyze data obtained from the inpatient unit that primarily cared for patients with hematologic malignancies, as these patients frequently have complex medical problems and are at particular risk for poor outcomes following development of critical illness. [23]

## Methods

### Setting and Study Population

This retrospective study utilized a cohort of adult patients hospitalized from 2009 to 2010 on an inpatient medical ward at the Ronald Reagan UCLA Medical Center, an academic tertiary medical center with 540 inpatient beds. The majority of these patients had leukemia, lymphoma and plasma cell dyscrasia as the primary diagnosis. In addition, the majority of these admissions were for chemotherapy, stem cell transplantation and neutropenic surveillance.

### Ethics

The study was reviewed and approved by the UCLA Institutional Review Board (IRB# 12–000482). The patient records were anonymized and de-identified prior to analysis.

### Neural Network Model

The features (predictors) used to develop the neural network model included all vital signs and laboratory studies for each patient during his/her hospitalization. Specifically, we analyzed systolic and diastolic blood pressures, heart rate, respiratory rate and temperature. We also incorporated white blood cell count, hemoglobin, platelet count, sodium, potassium, chloride, total CO2, BUN (blood urea nitrogen), creatinine and glucose into our model. These parameters were chosen as they are routinely obtained on all hospitalized patients and frequently monitored (i.e., at least daily up to several times a day). We did not include other laboratory studies such as liver function tests and coagulation studies as they are not routinely obtained in all hospitalized patients. For each admission, we included all the measurements of the vital signs and laboratory values until discharge for the control group and up until 4 hours prior to ICU transfer or cardiac arrest for the group that developed clinical deterioration. For the clinical deterioration group, we included measurements up to 4 hours prior to the clinical deterioration event. We did not include measurements closer than 4 hours because we felt that a warning system that gave at least a 4 hour warning window would allow clinicians sufficient time to act if the model indeed did work. Because laboratory studies are obtained at a lower frequency (typically once to twice a day) than vital signs, there were missing values. To deal with these missing values, we used the last observation carried forward. Otherwise, there was no further preprocessing of the data.

The entire cohort was randomly split up into the model-building cohort, cross-validation cohort and testing cohort. The random assortment was done by admissions and stratified by clinical deterioration so that the time series and time dependencies were preserved. The model-building cohort (comprised of 50% of admissions) was used to develop the neural network. The cross-validation cohort (comprised of 25% of admissions) was used to fine-tune the neural network parameters (number of hidden nodes and learning rate) and determine the number of interaction terms. After model building and optimization, the final model was then tested on the test cohort (comprised of 25% of admissions) to determine the model’s performance characteristics.

**Fig 1:**
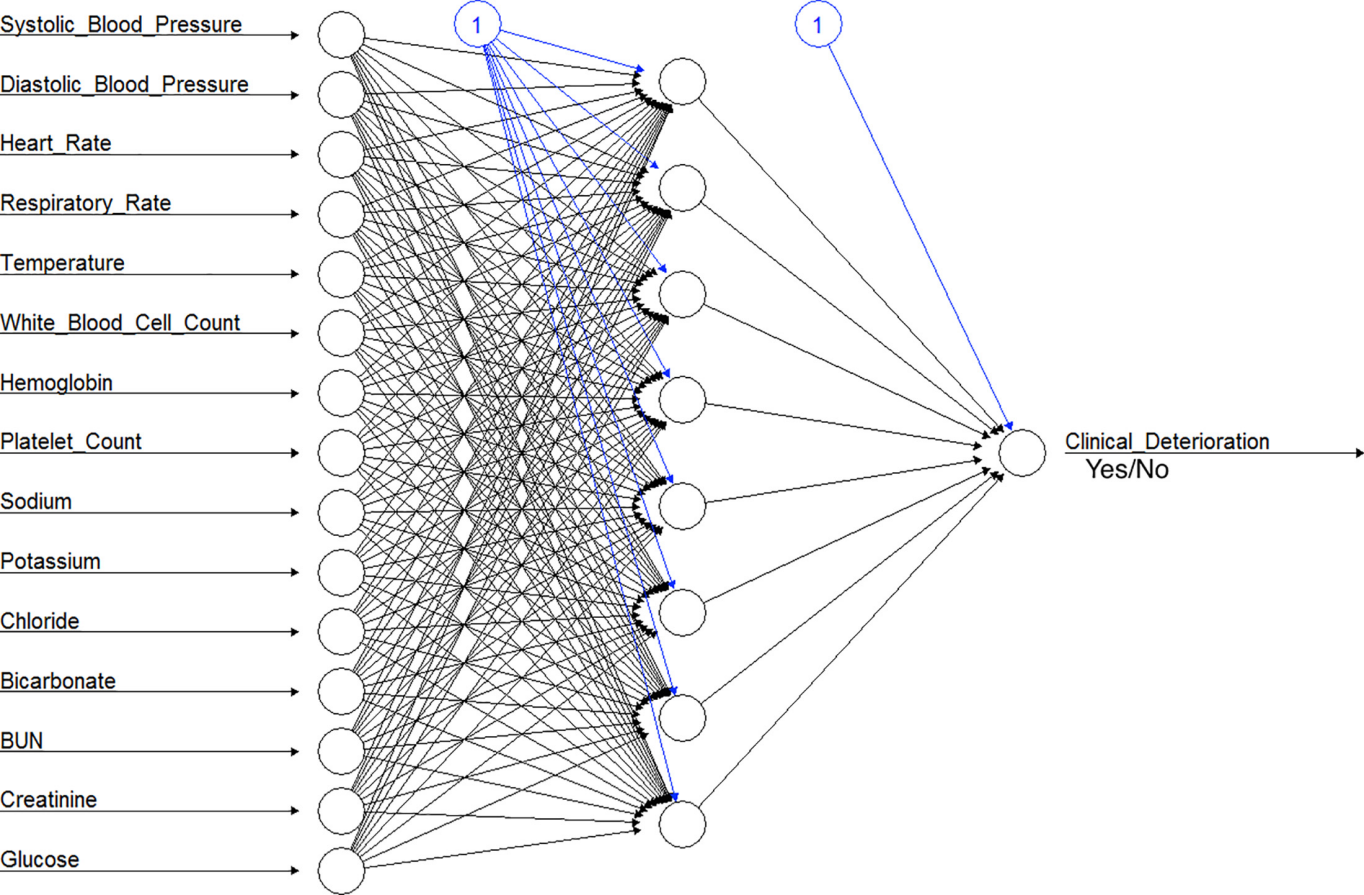
Representative neural network model demonstrating a simplified version of the neural network used to predict clinical deterioration in hematologic malignancy patients. The features (predictors) are listed on the left and represented by the circles which are the input nodes. The middle layer of circles represent the hidden layer with the circles representing the hidden nodes. The far right single circle represents the output node that serves to predict clinical deterioration from the neural network.

The neural network was built with 1 hidden layer (representative example shown in Fig 1). The weights of the neural network were determined by minimizing the mean squared error through gradient descent in a process called standard back propagation.[20] The number of hidden nodes and the learning rate were adjusted for optimal performance by testing the model on the cross-validation cohort. The features used as predictors were age, sex, vital signs and laboratory studies as listed above. Interaction terms were included with systolic blood pressure and the other features, heart rate and the other features, and respiratory rate and the other features. The R package RSNNS was used.[24]

### Test Statistics and Comparisons to Existing Models

After the neural network model was built and optimized, the test statistics were derived by applying the neural network model to the test cohort only. The test statistics did not include model performance on the model cohort or the cross-validation cohort. The test statistics we evaluated were the positive predictive value, area under the curve (AUC) from receiver operating characteristic (ROC) analysis and the unweighted F score. The ROC curve was generated using the ROCR package in R. [25]

Simulation was also performed on the cohort by randomly resorting the cross-validation and testing cohorts (i.e., 50% of subjects not used for model building). This was performed to generate a mean and 95% confidence interval. The random resorting was performed 100 times.

Comparison was made between our neural network model and the VitalPac Early Warning System (ViEWS) prediction model.[8] Our hospital did not have a specific protocol in place to record mental status (AVPU [alert, verbal, response to pain, unresponsive]) in our electronic record, as is used in the MEWS and ViEWS models.[8, 10] We also did not utilize oxygen saturation and whether the patient used supplemental oxygen. Therefore, in order for us to calculate the ViEWS score, we assigned the maximal score of 3 for mental status (AVPU) to subjects who developed clinical deterioration ("cases") and assigned a score of 0 for those that did not develop clinical deterioration ("controls"). Similarly, we assigned a maximal score of 3 for oxygen saturation to cases and a score of 0 to controls. Finally, the maximum score of 3 was assigned for the category of use of supplemental oxygen to those that developed clinical deterioration and a score of 0 to control subjects. We reasoned that imputing these values would artificially enhance the predictive value of ViEWS (i.e., provide the "best-case" scenario) when comparing the performance of ViEWS against our neural network. The range of positive predictive value reported for the ViEWS model was derived from choosing various cut-points on the ViEWS score to determine the range of sensitivity and specificity.

As an alternate method of dealing with the missing information of supplemental oxygen and AVPU scores, we performed simulation for these scores and then calculate the performance of the ViEWS score with this simulated data. The simulation was performed 100 times and the results are reported as a mean AUC with a 95% confidence interval.

## Results

### Demographics

Between 2009 and 2010, data were obtained from 565 consecutive admissions to the medical ward that primarily houses admitted adult patients with hematologic malignancies. Patient characteristics, cancer diagnoses and treatments are presented in Table 1. Forty-three admissions (7.6%) resulted in severe clinical deterioration (as defined by ICU transfers and code events) while 522 (92.4%) did not result in clinical deterioration (i.e., the control group). This is similar to other studies, where the rates of clinical deterioration events have been reported to range from 5–10% of admissions.[1, 8, 10, 15, 26] The control group and clinical deterioration group had comparable demographics with similar median ages, gender distribution, and median time to discharge or clinical deterioration. In addition, both groups had similar proportions of patients with AML and ALL, and rates of admissions for neutropenic surveillance and chemotherapy. The clinical deterioration group had more allogeneic stem cell transplant admissions while the control group had more autologous stem cell transplant admissions.

**Table 1:** Demographics.

Characteristic	Control Group	Clinical Deterioration Group	p-value
Number of admissions	522	43	
Median age (range) (in years)	52 (range: 17–95)	55 (range: 25–79)	0.2440
Percentage male	54.98%	55.81%	1.0000
Median time to discharge or clinical deterioration (days)	14.93	17.53	0.2019
**Cancer Diagnosis**			
AML	203 (38.89%)	20 (46.51%)	0.4118
ALL	58 (11.11%)	8 (18.60%)	0.2211
CML	13 (2.49%)	3 (6.98%)	0.2200
Myelodysplastic syndrome	13 (2.49%)	2 (4.65%)	0.7236
T-cell lymphoma/leukemia	18 (3.45%)	2 (4.65%)	1.0000
CLL	8 (1.53%)	1 (2.33%)	1.0000
Diffuse large B-cell lymphoma	47 (9.00%)	1 (2.33%)	0.2205
Hodgkin's lymphoma	22 (4.21%)	1 (2.33%)	0.8406
Malignant melanoma	5 (0.96%)	1 (2.33%)	0.9465
Amyloidosis, primary	2 (0.38%)	1 (2.33%)	0.5531
Aplastic anemia	20 (3.83%)	1 (2.33%)	0.9343
Biphenotypic leukemia	2 (0.38%)	1 (2.33%)	0.5531
Renal cell carcinoma	0 (0.00%)	1 (2.33%)	0.1096
Other cancer diagnoses	111 (21.26%)	0 (0.00%)	0.0015
**Treatments Received**			
Chemotherapy	207 (39.66%)	16 (37.21%)	0.8783
Allogeneic stem cell transplantation	64 (12.26%)	12 (27.91%)	0.0079
Neutropenic surveillance	88 (16.86%)	8 (18.60%)	0.9347
Autologous stem cell transplantation	90 (17.24%)	1 (2.33%)	0.0192
Other	73 (13.98%)	6 (13.95%)	1.0000

Other cancer diagnoses did not have any clinical deterioration and include seminoma, germinoma, gallbladder adenocarcinoma, Waldenstrom macroglobulinemia, sarcoma, and paraganglioma. Other treatments include immunotherapy and interleukin-2 therapy.

### Model Construction and Performance

The neural network was built on 50% of the combined cohort (565 admissions). Cross-validation was performed on a separate 25%. The cross-validation allowed further optimization of the neural network, resulting in a final neural network with 1 hidden layer, 24 hidden nodes and a learning rate of 0.01. The positive predictive value was then determined by applying the optimized final model on the separate 25% test cohort. The positive predictive value on the test cohort was 77.58% in contrast to previous models that report a typical positive predictive value of 5–10%.[10, 15] The negative predictive value was 99.19%, with sensitivity 93.33% and specificity 96.85%.

With prediction, the model frequently suffers when using data further away from the event of concern. Therefore, we examined how analyzing data up to earlier timepoints before clinical deterioration would affect model performance. We found that the performance of the neural network suffers only minimally when using only the data up to 8 hours prior to ICU transfer/cardiac arrest. The positive predictive value decreases to 76.47%, negative predictive value decreases to 98.4%, whereas sensitivity decreases to 86.67% and specificity remains at 96.85%. At 12 hours prior to ICU transfer/cardiac arrest the performance worsens, with a positive predictive value of 63.64% and negative predictive value of 93.89%. The sensitivity decreases to 46.67%, while specificity remains at 96.85%.

Table 2 reports the test statistics using simulation from random resorting of the cross-validation and training cohorts. The simulation process resulted in a positive predictive value of 81.98% [95% CI: 72.68–91.27%]. Overall sensitivity of the neural network was 84% [95% CI: 77% –91%], with a specificity of 98% [95% CI: 98%– 99%] and AUC of 0.92 [95% CI: 0.88–0.95].

**Table 2:** Test Performance from Simulation.

Positive predictive value (with 95% confidence interval)	81.98% [95% CI: 72.68% - 91.27%]
Sensitivity (with 95% confidence interval)	84% [95% CI: 77% - 91%]
Specificity (with 95% confidence interval)	98% [95% CI: 98% - 99%]
AUC (with 95% confidence interval)	0.92 [95% CI: 0.88–0.95]

### Model Comparison to ViEWS

We next wanted to compare the performance of our neural network based model against a widely used early warning scoring system. The VitalPAC Early Warning System is a widely adopted model for predicting risk of adverse events in admitted patients.[27, 28] We therefore examined the performance of the ViEWS model on our cohort of patients on the hematologic malignancy medical floor. Despite giving optimal scoring to the patients that developed clinical deterioration in the ViEWS model for mental status, oxygen saturation and use of supplemental oxygen, our neural network model performed better than the ViEWS model. (Table 3) The neural network positive predictive value had a range of 73–91% compared to 1–24% in the ViEWS model. The AUC for the neural network model ranged from 0.88–0.95 compared to the AUC of 0.69 using the ViEWS model. The F score was also better for the neural network model (0.81–0.85) compared to the ViEWS model (0.01–0.34).

**Table 3:** Comparison between Neural Network Model and VIEWS Model on Current Data.

	Neural network based model (95% Confidence Interval)	ViEWS
Positive predictive value	72.68–91.27%	1.14–23.89%
AUC	0.88–0.95	0.69
F score	0.81–0.85	0.01–0.34

Using simulation for missing information on supplemental oxygen and AVPU score, we ran 100 simulations and report the performance of the ViEWS score as the mean and 95% confidence interval. The AUC of the ViEWS model was 0.67 [0.65–0.69].

### Subset Analysis

Given that the positive predictive value depends upon the prevalence of clinical deterioration, we analyzed how our model performed in subsets of patients with varying rates of clinical deterioration. The prevalence of clinical deterioration is highest in patients admitted for allogeneic stem cell transplantation, whereas among patients admitted for autologous stem cell transplantation, the rate of clinical deterioration is much lower. Table 4 represents the positive predictive value of the neural network if the same test statistics were applied to the different hematologic malignancy subpopulations. Not surprisingly, the positive predictive value was lowest for patients admitted for autologous stem cell transplantation. However, the model generally maintained high performance across patients at average and higher risk for clinical deterioration.

**Table 4:** Comparison of Positive Predictive Value in Different At-Risk Populations Using the Neural Network Based Predictive Model.

	Positive Predictive Value from Neural Network Model	Percentage of Patients that Developed Clinical Deterioration
All patients from hematologic malignancy ward	77.58%	7.61%
Patients admitted that were treated with chemotherapy	76.45%	7.17%
Patients admitted for allogeneic stem cell transplantation	88.73%	15.79%
Patients admitted for autologous stem cell transplantation	31.82%	1.10%
Patients admitted for neutropenic surveillance	79.25%	8.33%

## Discussion

In this study, we created a type of neural network-based model using multilayer perceptron training, to determine whether this approach could predict significant clinical deterioration events in a cohort of admitted patients with hematological malignancies. The neural network was "trained" using routinely available clinical data (e.g., age, gender, and all of the vital signs and labs obtained during hospitalization) that was input from our EMR. We conjectured that a NN-based model would be able to discern complex patterns, such as changes in heart rate variability, that would forecast clinical deterioration more accurately than one of the best-performing early warning scores (ViEWS). To our knowledge, we are the first to utilize a neural network (multilayer perceptron) based model for the prediction of clinical deterioration on hospitalized patients, utilizing routinely obtained vital signs and laboratory studies. While this is the first step, we have demonstrated the powerful potential of applying machine learning techniques to the vast amount of data recorded in EMRs, which in our hands were able to predict significant clinical deterioration events with high accuracy using data up to 4 hours before the event. Ultimately, if successful, neural networks could be incorporated into hospital EMR systems in real-time to provide ongoing surveillance of inpatient clinical data using more sophisticated pattern recognition analysis, which would provide clinicians with more accurate forecasts of clinical deterioration.

This study was based on a fairly well-defined cohort of hospitalized patients with primarily hematologic malignancy, and was able to perform with a positive predictive rate of 77.58% on an independent testing cohort, an improvement above that previously reported in literature of approximately 10%.[10, 15] The improved performance of this neural network based model is likely based on the ability of the neural network to discriminate non-linear patterns.[20] Furthermore, accurate forecasting of clinical deterioration at an early enough timepoint where therapeutic intervention can have a meaningful impact on outcomes is challenging, even for the most experienced of clinicians. Our study was able to predict events using data up to 4 hours before the event, which would provide time for interventions such as additional testing, and administration of IV fluids, antibiotics, and other therapies. Future studies will examine how well different neural network approaches perform at earlier times before the event (e.g., 8 and 12 hours prior to the event).

The purpose of designing a predictive model for clinical deterioration should not be to add to the noise of existing alarms that already have low positive predictive values. Rather, they should integrate these existing alarms into a single alarm that can be trusted to give a reliable alarm. Another advantage of a higher positive predictive value (or lower false positive rate) is that there would be fewer false alarms to react to for a rapid response team, reducing clinical workload and potentially reducing costs. Therefore, the major advantage of our neural network model is the improved positive predictive value. We focused on positive predictive value because it is a more clinically useful test statistic for clinicians as it integrates information about prevalence and test performance. The higher positive predictive value allows the clinician to be more confident in the alert when it is sounded as compared to other models where the positive predictive value is on the order of 10%.[10, 15] Increased false alarm alerts result in alarm fatigue and alarm desensitization, which decreases the likelihood of a timely response to the alarms.[14, 17, 29–31] Therefore, a system that predicts clinical deterioration should be focused on a higher positive predictive value. The importance of a higher positive predictive value is supported by studies suggesting that the response rate to an alarm is correlated to the perceived reliability of the alarm.[14, 32–34] Therefore, an alarm with a low positive predictive value would be responded to less frequently that one with a higher positive predictive value.

Pinsky et al have studied the use of an integrated monitor (Visensia OBS Medical) that integrates information from heart rate, respiratory rate, blood pressure, oxygen saturation and temperature to detect cardiorespiratory instability in step down units.[35, 36] They reported a sensitivity of 70.5% and a specificity of 71%. Given the higher prevalence of cardiorespiratory instability on these floors (between 25–34%), it would be expected that the positive predictive value would be between 44.76–55.60%.[36, 37] The Visensia monitor is a probabilistic monitor that quantifies what is normal (Gaussian) and then identifies cardiorespiratory instability when the vital signs that are fed into it lie outside of a proprietary definition of normal. In a sense, this is a form of anomaly detection, much like what industry uses to determine a defective part in manufacturing or what credit card companies use to find credit fraud. We have also studied the use of an anomaly detection system in the form of a multivariate Gaussian model but found that approach did not perform as well as the neural network (data not shown). The higher prevalence of clinical deterioration in the step-down units noted by Pinsky et al suggests that if our neural network system were optimized to work in a step-down unit, the higher prevalence would lead to an even higher positive predictive value.

While we wanted to build a prediction model solely based on routinely obtained vital signs and laboratory values, Table 4 suggests that the inclusion of additional clinical information would improve the model. For instance, we expect that patients being admitted for allogeneic stem cell transplantation have a higher prevalence of clinical deterioration because of the intensity of the treatment as compared to patients being admitted for autologous stem cell transplantation, as we expect that they will engraft more readily and not develop graft-versus-host disease. As electronic medical record systems start to incorporate such a priori clinical data more readily, they will be included in the prediction model to better refine the model for different population cohorts. Furthermore, the predictors for the neural network would be readily scalable. If, for example, a non-invasive method for determining cardiac output were more readily available, this would be easily incorporated into the neural network model, after sufficient training. Hence, this approach provides enhanced flexibility to customize the warning system to specific patient populations.

One potential limitation of our study is that it was not developed and tested on a generalized hospital population but rather a more defined population of patients that were primarily admitted with a hematologic malignancy diagnosis. While this was a more focused study, the prevalence of clinical deterioration is similar to other studies.[1, 8] However, this may also highlight the importance of condition- or patient-acuity specific algorithms. Instead of developing an overall model for the entire hospital, the performance of the prediction models may be improved by developing different models for different hospital wards, as the type of patient or nursing care may vary depending on the ward that the patient was admitted to.

There is the potential for overfitting with any model, however we tried to deal with this by setting aside a random cohort only for testing which was not used in model development. In addition, we report the results of simulation/cross-validation as an estimate of the confidence interval of the test statistics. It will depend on future study to further validate the results here.

Another limitation common in machine learning algorithms is that the prediction system becomes a "black box" and does not lend itself to easy interpretation of physiology, as compared to prediction systems built on methods such as linear regression. While a linear prediction system might be more understandable (e.g., that a certain decrease in systolic blood pressure would lead to a higher chance of clinical deterioration), the neural network is not able to provide such interpretation. We would argue, however, that a busy clinician would readily trade away interpretability for the convenience of a more reliable warning system. In addition, this system might inform the clinician as to which patients might require more attention, allowing them to prioritize their clinical efforts.

One important issue that remains unaddressed is whether earlier prediction of clinical deterioration will result in earlier interventions that improve overall outcomes. The creation of rapid response teams is an example of a systematic, early intervention that has been implemented in an attempt to improve clinical outcomes, but has had variable results. Certain patients may simply have a fixed trajectory, where any intervention may not alter the course of illness. However, other therapies, such as prompt initiation of antibiotics in patients with pneumonia, achievement of anticoagulation goals, and aggressive hemodynamic resuscitation in patients with early sepsis have demonstrated that timing of therapy remains a critical factor in many clinical situations. Hence, we believe that clinicians and patients will benefit from early warning systems that are accurate, although formal studies are necessary to determine the magnitude of this benefit. Once we have optimized our model, we anticipate initiating a clinical trial where we examine whether our neural-network based warning system leads to earlier intervention, decreases number of ICU transfers and cardiopulmonary arrests, and improves other clinical outcomes compared to current standard practice.

## Conclusion

We have demonstrated the ability of using a neural network model to predict clinical deterioration in a cohort of hospitalized patients that were primarily admitted for hematologic malignancy diagnoses. The neural network based model performs with a higher positive predictive value compared to other existing models and accomplishes this by employing routinely collected vital signs and laboratory values, precluding the need for specialized training of staff in calculating early warning scores.
